# Magneto-Responsive Textiles for Non-Invasive Heating

**DOI:** 10.3390/ijms241411744

**Published:** 2023-07-21

**Authors:** Arkadiusz Józefczak, Katarzyna Kaczmarek, Rafał Bielas, Jitka Procházková, Ivo Šafařík

**Affiliations:** 1 Faculty of Physics, Adam Mickiewicz University in Poznań, Uniwersytetu Poznańskiego 2, 61-614 Poznań, Poland; katarzyna.kaczmarek@amu.edu.pl (K.K.); rafal.bielas@amu.edu.pl (R.B.); 2 Department of Nanobiotechnology, Biology Centre, ISBB, Czech Academy of Sciences, Na Sádkách 7, 370 05 České Budějovice, Czech Republicivosaf@yahoo.com (I.Š.); 3 Regional Centre of Advanced Technologies and Materials, Czech Advanced Technology and Research Institute, Palacký University, Šlechtitelů 27, 783 71 Olomouc, Czech Republic

**Keywords:** smart materials, magnetic textiles, magnetic nanoparticles, magnetic hyperthermia, tissue-mimicking phantom

## Abstract

Magneto-responsive textiles have emerged lately as an important carrier in various fields, including biomedical engineering. To date, most research has been performed on single magnetic fibers and focused mainly on the physical characterization of magnetic textiles. Herein, from simple woven and non-woven textiles we engineered materials with magnetic properties that can become potential candidates for a smart magnetic platform for heating treatments. Experiments were performed on tissue-mimicking materials to test the textiles’ heating efficiency in the site of interest. When the heat was induced with magneto-responsive textiles, the temperature increase in tissue-mimicking phantoms depended on several factors, such as the type of basic textile material, the concentration of magnetic nanoparticles deposited on the textile’s surface, and the number of layers covering the phantom. The values of temperature elevation, achieved with the use of magnetic textiles, are sufficient for potential application in magnetic hyperthermia therapies and as heating patches or bandages.

## 1. Introduction

Materials that are designed in a way to have their properties significantly changed in a controlled fashion by external stimuli, i.e., light, electric or magnetic field, stress, and pH change, are called smart materials. Textiles that are responsive to various stimuli have gained significant scientific and application interest in the last decade. According to reports, the growth of the stimuli-responsive textile industry and research in this field is exponential [1]. One of the intensively researched sectors is so-called *textronics* and refers to electronic devices that are wearable and advanced in terms of their integration into the textile structure [1]. Such smart materials could be used in our daily life, for instance, as a part of personal health-monitoring sensors [2] or to deliver a heat on demand to the site of interest. One of the approaches to locally inducing temperature elevation in a controllable way is the generation of heat through magnetic heating. In this case, the magnetic energy is dissipated in the form of heat due to relaxation or/and hysteresis processes in magnetic materials exposed to time-varying magnetic fields, commonly an alternating (AC) magnetic field [3]. The use of such induced temperature elevation, either to supplement conventional cancer treatment [4] or to directly damage cancer cells [5] has been investigated thoroughly in recent decades and is currently being introduced as a clinical procedure [6].

Magnetic heating has already been partially investigated in the field of responsive textiles. Amarjargal et al. showed the ability of a magnetite particle-coated nanofibrous membrane, which was fabricated using the electrospinning technique, to generate heat under AC magnetic fields [7]. When discussing the obtained temperature increase, authors suggested that magnetic nanoparticles (MNPs) were firmly anchored into the fibers, and therefore the Brownian relaxation heating mechanism was limited. Other scientific groups also suggested the immobilization of MNPs within the textile structure that deteriorates the heating performance of magnetic textiles [8,9]. Such behavior is similar to the MNPs being entrapped in a gel structure of agar phantoms [10]. The use of textiles as carriers of magnetic materials brings novel opportunities for modern treatments. One of their advantages is the versatility of textile materials to be functionalized and customized with desired materials to create a theranostic platform, i.e., the paradigm of combining therapy and diagnostic modalities into one carrier [11]. GhavamiNejad et al., proposed MRI-guided fibers capable of pH-dependent chemo-therapeutic drug release in the environment of cancer tissue and magnetic heating generation [12]. In many cases, the repeated application of the anti-cancer treatment, including local magnetic heating, is necessary due to the chance of tumor metastasis. In actual clinical practice, carriers must maintain their heating ability for several heating and cooling cycles. The use of magnetic textiles directly attached to the tissue of interest is expected to be a much more effective way of delivering therapeutic agents, as the circulation time of MNPs for conventional intravenous injection is not long enough [13]. Moreover, because the possibility of washing particles out of textiles is limited, the high loading capacity of the magnetically modified textiles allows for their efficient interactions with tumors and evenly distributed temperature during the therapy when an alternating magnetic field is applied [14].

To date, most of the results from magnetic heating experiments have concerned different magnetic materials such as fibers, mats, and membranes without paying attention to the target medium. The temperature increase induced by magnetic energy losses has been commonly investigated in water suspensions or the air [7,9,12,15]. Only a few works reported experiments performed on the textiles placed in actual tissues [8] or agar gel [16]. However, it should be noted that agar gel was only used to fix the research sample in one place and avoid sample movement during experiments. The temperature increase was not analyzed in regard to the medium’s properties. To the best of our knowledge, magnetic textile materials have not been used in in vitro experiments for magnetic heating of tissue-mimicking phantoms to test the textiles’ heating efficiency at the site of interest. The ability of magnetic material to generate heat, at a sufficient rate, is not the only factor affecting the final temperature increase inside, e.g., simulated cancer tissue. In the case of magnetic textiles applied to the tissue or tissue-mimicking phantom, heat transfer also plays a crucial role. Therefore, we investigate the temperature elevation in agar-based tissue-mimicking phantoms caused by the heating of surface-modified magnetic textiles.

Magnetic textiles consisting of magnetic fibers fabricated by the addition of magnetic fluid prior to the formation of polymer fibers (polyvinyl butyral PVB) in the process of electrospinning were tested previously [17]. Although the potential application of such textile, either in magnetic hyperthermia therapy for internal tumors or as implants for heating at the skin’s surface, was suggested, a more robust and efficient method of loading of magnetic materials onto the textiles can be used to efficiently provide the heating effect on the phantoms. The method of preparation of smart textiles is crucial when it comes to the efficiency of magnetic heating. Matos et al. [18] showed the improved heating ability of magnetic textiles prepared by the incorporation of MNPs into a fibrous structure, compared to ones fabricated by a surface deposition of magnetic fluid onto the already formed textile materials. In a similar way, we prepared textiles used in this study. Commercially available textiles acquired their magnetic properties through deposition of magnetic iron oxide (nano)particles on their surface. Such prepared textiles can be used as a coating layer for phantoms to generate temperature increase inside the phantom. In this work, we used woven and non-woven textiles loaded with the same type of magnetic nanoparticles. Subsequently, we investigated the heating efficiency in agar-based tissue-mimicking phantoms for several scenarios. We researched the heating effect of textile multilayers and the synergetic effect of heat transferred into the phantom by magnetic nanoparticles embedded in a textile and contained inside phantoms. The determined heating efficiency was compared to the characteristics of materials to show that simple woven and non-woven textile materials can become potential candidates for a smart magnetic platform for hyperthermia treatment or therapy.

## 2. Results

### 2.1. Properties of Magnetic Nanoparticles and Magnetic Textiles

**Figure 1:**
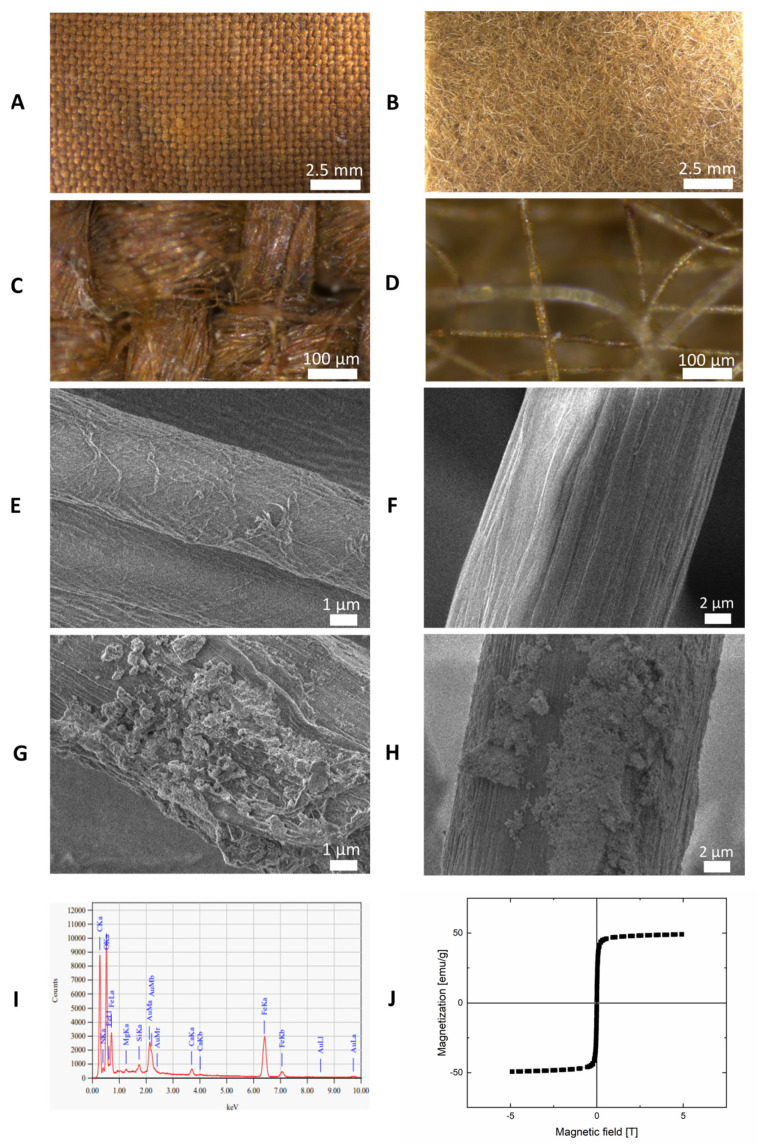
Characterization of magnetic textiles used in the experiments. Optical microscopy images of two types of magnetic textiles used in the study with different magnifications: textile MG TEX1 (**A,C**) and textile MG TEX3 (**B**,**D**). Scanning electron microscopy (SEM) images of single fibers extracted from non-magnetic TEX1 (**E**) and non-magnetic TEX3 (**F**). SEM images of single fibers extracted from MG TEX1 (**G**) and MG TEX3 (**H**). Energy dispersive spectroscopy (EDS) analysis of single fiber extracted from MG TEX3 (**I**). Magnetization curve from VSM for nanoparticles used for doping textiles and phantoms (**J**).

Optical microscopy was used to present the structure and differences in the fiber alignment for woven (TEX1) (Figure 1A,C) and non-woven (TEX4) textiles (Figure 1B,D). Scanning electron microscopy (SEM) of native and modified textiles was employed for more detailed characterization. If we compare SEM images of fibers for a native textile (Figure 1E,F) with the structure of a magnetic textile (Figure 1G,H) we clearly see the presence of iron oxide nanoparticles and their aggregates on the surface of modified fibers. Most nanoparticles prepared by microwave-assisted synthesis had diameters between 50 and 150 nm, which is in accordance with a previous study [19]. However, micrometer-sized aggregates are also visible. The successful deposition of iron oxide nanoparticles on the modified textiles was also confirmed by energy-dispersive X-ray spectroscopy. Typical iron peaks at 0.705, 6.398, and 7.057 keV were present (Figure 1I). Figure 1J presents the magnetization curve of magnetic nanoparticles used in our study. The graph shows that nanoparticles exhibit high magnetization saturation and a very small hysteresis loop proving their superparamagnetic properties.

### 2.2. Heating Ability of Magnetic Textiles

**Figure 2:**
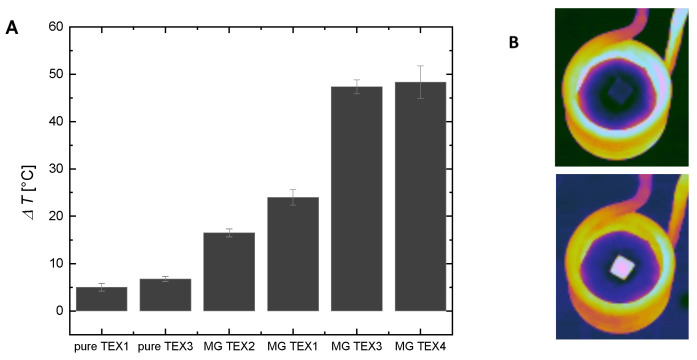
Comparison of the heating effect of different magnetic textiles used in the study. (**A**) Temperature elevation recorded with an IR camera for the 60th second of measurement for the center of the textile sample. (**B**) IR image for pure textile and magnetic textile during magnetic hyperthermia. Magnetic textiles changed color as their temperature increased. The magnetic field intensity was 16.2 kA/m, and the frequency was 356 kHz.

Magnetic hyperthermia experiments were performed on pure non-magnetic (native) and magnetic textiles. Textiles were placed inside the geometrical center of the magnetic coil with a thermometer probe underneath. As can be seen in Figure 2, we observed a slight temperature rise for native textiles. However, this increase is not caused by any of the magnetic mechanisms, as there was no magnetic material in the textile present. It was caused by the heat generated by a working magnetic coil under high electric current. The temperature increase registered for magnetic textiles was significantly higher (up to 10 times) than the temperature elevation registered for pure textiles. The highest temperature increase was observed with an acrylic felt-base textile (MG TEX4 and MG TEX3) and the smallest was observed with a cotton textile MG TEX2. The obtained results agreed with the content of magnetic iron oxide nanoparticles inside textiles as presented in Table 1, and proved that the functionalization of magnetic textiles with magnetic material was successful. We fabricated smart magneto-responsive textiles that generate heat in response to external alternating magnetic fields.

### 2.3. Magnetic Heating of Pure Tissue-Mimicking Phantoms

Once we proved that magnetic textiles generate heat, we performed experiments to research the ability of textiles to transfer heat into other materials. Agar-based tissue-mimicking phantoms were used and covered with layer/s of non-magnetic and magnetic textiles.

#### 2.3.1. Single Layer of Textiles

**Figure 3:**
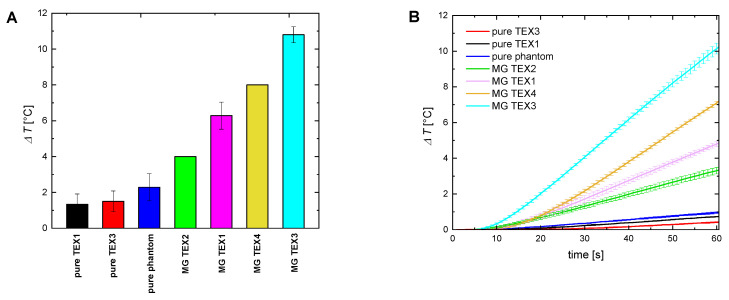
Comparison of temperature elevation induced inside the pure agar-based tissue-mimicking phantom by different (pure and magnetic) textiles used in the study. (**A**) Temperature elevation from the IR image for the last second (60th second) of magnetic heating for the center of the phantom. (**B**) Temperature elevation vs. time measured with an optical thermometer for the center of the phantom. The magnetic field intensity was 16.2 kA/m, and the frequency was 356 kHz.

Firstly, we performed magnetic hyperthermia experiments on pure agar tissue-mimicking phantoms covered with a single layer of textile. Temperature rise was recorded with the IR camera (Figure 3A) and optical sensor (Figure 3B) placed inside the phantom. Temperature for agar phantoms not covered with any type of textile slightly increased, similar to agar phantoms covered with non-magnetic textiles. As we described in paragraph 3.2, such temperature rise does not come from a magnetic mechanism, but it is a result of heat generated by a working magnetic coil. Interestingly, non-covered agar phantoms exhibited higher temperature increase compared to agar coated with non-magnetic textiles. We assume this is due to the textile becoming an isolator and a barrier protecting the agar phantom from heat transferred from the coil. Phantoms covered with magnetic textiles achieved noticeably higher temperature increases due to the activation of heating mechanisms of magnetic particles in the alternating magnetic field. The highest temperature rise was observed in acrylic felt textiles (MG TEX3 and MG TEX4) and smaller ones were observed in cotton-based MG TEX1 and MG TEX2. Although MG TEX3 and MG TEX4 contained the same amount of magnetic material inside (2.62 mg/cm^3^), MG TEX3 gave a higher temperature increase compared to MG TEX4. It is worth mentioning that MG TEX4 was fabricated by a Thermo Press device, so it was thinner compared to MG TEX3. As in the case of non-magnetic textiles playing the role of heat isolators, here, the thicker magnetic textile (MG TEX3) prevented acquired heat from dissipating. In other words, a thicker layer of textile allowed for heat generated by the magnetic textile to accumulate inside the phantom, which subsequently caused a higher temperature increase.

The tendency of temperature increase is similar to optical probe measurements (Figure 3B). The highest temperature increase was obtained with the magnetic acrylic felt MG TEX 3 and MG TEX 4 and smaller increases were obtained with the magnetic textiles MG TEX1 and MG TEX2. It is worth remembering that the temperature values obtained with IR thermometry and optical probes are slightly different as they show temperature rises in different places. The IR thermometer shows the temperature of the top surface of the agar phantom, and the optical probe shows the temperature inside a phantom.

#### 2.3.2. Multilayer of Textiles

**Figure 4:**
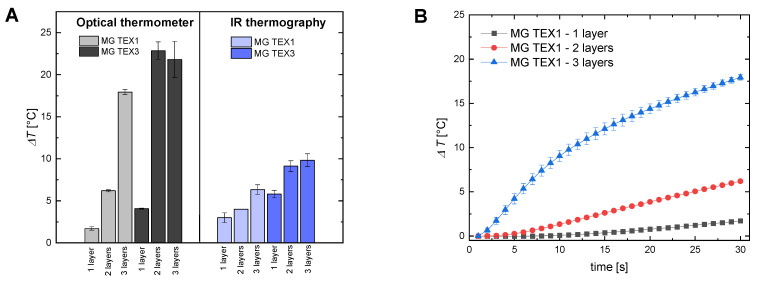
Comparison of temperature distribution inside the tissue-mimicking phantom covered with multiple layers of different magnetic textiles used in the study. (**A**) The temperature increase recorded with an optical fiber and IR camera, for the 30 s of measurement for various number of textile layer of MG TEX1 and MG TEX3 and (**B**) temperature change vs. time measured with optical thermometer for the center of the phantom for magnetic textile MG TEX1. The magnetic field intensity was 16.2 kA/m, and the frequency 356 kHz.

Secondly, we performed magnetic hyperthermia experiments on the agar tissue-mimicking phantoms covered with multiple layers of textile. Temperature rise was recorded with the optical sensor centrally placed inside the phantom and the IR camera. Here the experiment lasted 30 s (instead of 60 s) due to rapid temperature increases. Figure 4A compares the final temperature rise recorded with an optical fiber and IR camera, for the 30 s of measurement for various numbers of textile layers of MG TEX1 and MG TEX3. Figure 4B presents detailed temperature increase as a function of time for the cotton-based magnetic textile MG TEX1. Based on Figure 4A, one can see that regardless of the method of temperature registration, three layers of cotton-based magnetic textile MG TEX1 gave the highest temperature increase in tissue-mimicking material, compared to two MG TEX1 layers or one MG TEX1 textile layer. Covering the tissue-mimicking phantom with three layers or two layers of MG TEX3 also gave a higher temperature increase compared to only one MG TEX3 layer. Interestingly, we did not observe here a significant difference between three and two textile layers. We assume that the lack of significant difference between two and three layers of acrylic felt MG TEX3 comes from the textile thickness (2 mm) preventing efficient heat transfer between textile layers. The phantom covered with one layer of MG TEX3 had a diameter of 14 mm, that with two layers had a diameter of18 mm, and that with three layers had a diameter of 22 mm compared to the diameter of 13 mm, 12 mm, and 11 mm for a phantom covered with three layers, two layers, and one layer of MG TEX1, respectively.

### 2.4. Synergistic Heating of Magnetic Textile and Magnetic Nanoparticles

**Figure 5:**
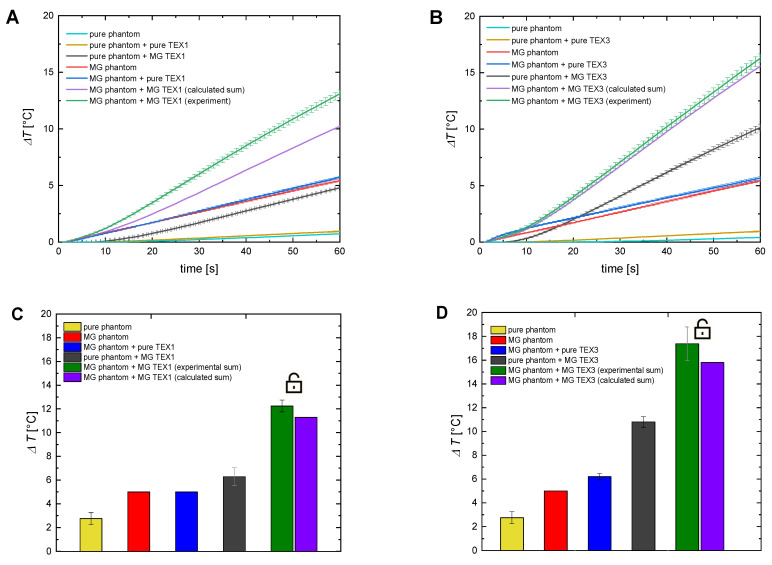
Comparison of the temperature elevation in the pure tissue-mimicking phantoms and the tissue-mimicking phantoms doped with nanoparticles for cotton-based TEX 1 (**A**) and acrylic felt textile TEX3 (**B**) for optical fiber, and (**C,D**) for the IR camera. The symbol of a locker symbolizes the unlocking of the Brownian relaxation mechanism. The magnetic field intensity was 16.2 kA/m, and the frequency was 356 kHz.

Once we proved the ability of magnetic textiles to generate heat that can be transferred into the agar-based phantoms, we decided to add magnetic nanoparticles (same as the one used for textile functionalization) during the preparation process into agar tissue-mimicking phantoms. Figure 5A,B present magnetic hyperthermia results for the optical probe, and Figure 5C,D present results for the IR thermography conducted on the non-covered pure and magnetic phantoms, and phantoms covered with non-magnetic and magnetic textiles.

Based on the presented results, it is clear that the addition of magnetic nanoparticles into pure agar phantoms increases their temperature. Similarly, the modification of pure textiles with magnetic nanoparticles gives rise to their magneto-responsiveness. The observed temperature increase for pure agar phantoms covered with magnetic textiles is higher than those covered with pure textiles. Hyperthermia experiments for magnetic phantoms covered with magnetic textiles resulted in the highest temperature increase. Here, the temperature increase comes from the heat generated by two sources, namely, MNPs placed inside phantoms and MNPs embedded in textiles. Interestingly, the experimental value of heat generated by the combination of magnetic hyperthermia from magnetic phantoms and magnetic textiles is larger than the calculated sum of those two sources of heat. We assume that this synergistic effect resulted from unblocking Brownian relaxation in tissue-mimicking materials with heat generated by magnetic textiles. Magnetic nanoparticles embedded in the structure of the gel are partially locked. Therefore, the Brownian mechanism responsible for heat generation is quenched. However, once the temperature of the tissue-mimicking phantom is increased, due to heat transfer from magnetic textiles, the size of the pores of the gel structure increases, leaving more space for particles to move, which is a reason for unblocking the Brownian relaxation mechanism [20]. In terms of potential applications, not only the magnitude of the heating effect is crucial, but also the rate of temperature increase. Figure S2 presents the comparison of the temperature increase rate (d*T/*d*t*) for all scenarios considered in our paper.

### 2.5. Magnetic Heating as a Function of Radial Distance from the Textile

**Figure 6:**
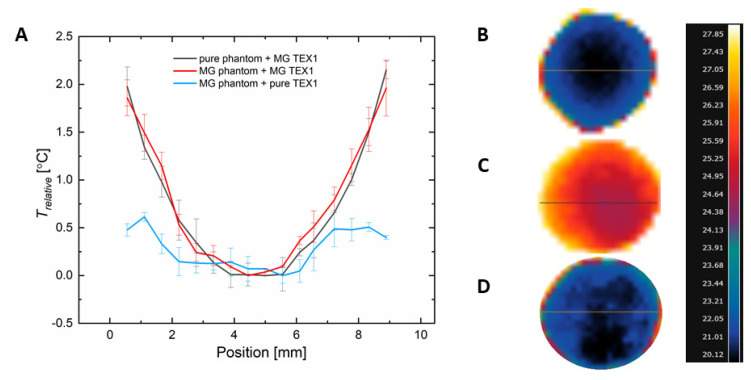
Comparison of spatial temperature distribution along a chosen cross-section in pure tissue-mimicking phantom covered with magnetic textile (MG TEX1), magnetic phantom covered with pure textile (TEX1), and magnetic phantom covered with magnetic textile (MG TEX1). (**A**) Relative temperature increase vs. position of pixels along the phantom’s diameter. The IR images of pure tissue-mimicking phantom covered with MG TEX1 (**B**), magnetic phantom covered with MG TEX1 (**C**), and magnetic phantom covered with pure woven TEX1 (**D**). The magnetic field intensity was 16.2 kA/m, and the frequency 356 kHz. The temperature values were obtained from the IR images for the last second of measurement (60th second). The IR images were taken from the top view of the phantom.

The results presented so far concern the temperature elevation observed in the center of the tested phantom either from the inside or from the top surface of the phantom’s body. However, it is expected that the temperature increase inside the phantom is inhomogeneous due to heat transfer. Therefore, we analyzed our data to present results as a function of the distance from the center of the phantom along its diameter. Figure 6 presents the IR results of the temperature increase in the phantom for three different scenarios, i.e., for a pure agar phantom covered with a magnetic textile (MG TEX1), a magnetic phantom covered with a pure textile (TEX1), and a magnetic phantom covered with a magnetic textile (MG TEX1). The presented results constitute the difference between the temperature value for each pixel along the cross-section line and the minimum temperature recorded for each case in the center of the phantom.

Interestingly, based on Figure 6A, when the temperature increase within the phantom’s body is relative to the minimum value, the trend of the temperature along the phantom diameter is very similar for the pure and magnetic phantoms covered with MG TEX1. This proves that the presence of magnetic nanoparticles inside the phantom does not disturb the heat transfer that comes from the textile. The deviation in the temperature for phantoms doped homogeneously with nanoparticles is small as expected. Figure 6B shows that the heat transfer arising from the magnetic textile takes place radially. The temperature increase is the highest in the vicinity of the magnetic textile and is the lowest in the center of the phantom, a radially furthest point from the textile. Figure 6D presents the temperature increase arising from magnetic nanoparticles alone. Here, the temperature does not rise radially, but due to overall nanoparticle distribution in the phantom, rises more uniformly throughout the phantom. Figure 6C shows the highest temperature increase as it increased from magnetic nanoparticles embedded in the phantom and in the textile. Nevertheless, the temperature rise decreases radially, with the coldest place located in the center of the phantom.

## 3. Discussion

The interest in using single nanofibers, magnetic mats, and textiles in biomedicine, including theranostic applications, has significantly increased over recent years [13,21,22,23]. Our proposed work fits in well with this trend and provides data for further field development. In this paper we presented a simple, facile, easy-to-scale-up method of designing woven and non-woven magneto-responsive textiles, by immersing or dripping them with a magnetic nanoparticle solution. We discussed their characteristics, properties, and possible future applications.

Therapeutic effects of magnetic hyperthermia occur when the physiological body temperature is increased by several Celsius degrees, up to 43–46 °C [3]. The values of temperature elevation (up to 11 °C for one textile layer, Figure 3) achieved with the use of proposed magnetic textiles are sufficient for potential application in magnetic hyperthermia therapies. What is important for future hyperthermia applications is that the temperature increase depends on the type of textile material, its thickness, the concentration of MNPs deposited on the textile’s surface, and the number of textile layers used. In our work, the highest temperature elevation was observed for the thickest, non-cotton textile embedded with the highest content of MNPs. Moreover, if we add nanoparticles into regions of interest, e.g., tissue coated with a magneto-responsive textile, the synergistic heating effect is observed. This behavior presents us with the opportunity of improving the heating performance by combining the heat induced internally and externally. As our experiment was designed to test agar-based samples using a magnetic coil surrounded with air, the heat transfer between our samples and the surrounding medium existed. It resembles the scenario of when magnetic textiles would deliver heat to the external region of a patient’s body, e.g., skin cancer. Therefore, the heat transfer should also be taken into account whilst considering future applications.

There exist more sophisticated methods of fabricating magnetic textiles than ours, such as electrospinning, where one can control the formation process, or obtaining modified fibers in adequate quantities, e.g., using sonochemical methods as presented in [24] for applications in catalysis. However, from the perspective of hyperthermia therapy efficiency, it is more important to use a smart material and take advantage of a robust method of fabrication, and provide a fast heat release to the region of interest. This could be more beneficial than developing nanofibers with a fine structure, which is evidence of an advancement in nanotechnology that cannot yet provide such a high thermal dosage to the tissues.

## 4. Materials and Methods

### 4.1. Preparation of Magnetic Textiles

As templates for magnetic textile (MG TEX) materials, we used two types of commercially available cloths, which differed in their internal structure. A cotton textile (white, fine plain-woven canvas, with a specific weight of 160 g m^−2^) was obtained locally. Before use, it was boiled three times in distilled water and air-dried. A nonwoven textile (Bastelfilz, with a specific weight of 150 g m^−2^ in the form of white 100% acrylic felt) was obtained from Max Bringmann KG, Wendelstein, Germany. To prepare magnetic nanoparticles (MNPs) suitable for textile functionalization, FeSO_4_·7H_2_O and NaOH were used as obtained from Sigma-Aldrich Co., St. Louis, MO, USA. The microwave-synthesized magnetic iron oxide particles were prepared as described recently [19]. Their characterization by electron microscopy and X-ray diffraction (XRD) analysis have confirmed that they are magnetite crystals [25,26]. To produce a magnetic cotton-based textile MG TEX1, the textile material was placed on a flat, non-adsorbing (glass) surface. 10% solution of FeSO_4_·7H_2_O was evenly dripped on the textile in the amount of 25 µL per cm^−2^. Then, the textile was air-dried at 40 °C for 3 h. The dried textile was transferred to a beaker containing 1% NaOH solution, placed in a microwave oven, and heated for 10 min at a power of 700 W. The textile with bound magnetic iron oxide particles was repeatedly washed with water and air-dried. The whole procedure was repeated three times. The cotton-based MG TEX2 was fabricated similarly to MG TEX1. The only difference was that the pristine textile material was immersed in the ferrous sulfate solution for 15 min. Acrylic felt-base magnetic textiles no. 3 (MG TEX3) and 4 (MG TEX4) were prepared using MNP suspensions prepared through microwave synthesis. After thorough washing with water and particle sedimentation, a particle suspension of 1:4 (volume of particles/total volume) was prepared. After 15 min of saturation, the modified textiles were placed on a flat glass surface and air-dried at 40 °C for at least 3 h. The textiles were repeatedly modified three times. In the case of MG TEX4, the steps were identical; additionally, it was modified by pressing in a dedicated device (Thermo Press provided by Rea, Šicí Technika Brother s.r.o., Prostějov, Czech Republic) at 200 °C for 90 s. The concentration of magnetic iron oxide particles per 1 cm^2^ of the textile materials was calculated from the mass differences of native and magnetically modified textile materials divided by their area measured in square centimeters. All magnetically modified textile samples exhibited a brown color. The binding of MNPs to the textile was strong, and almost no leakage of particles was observed during water treatment. Essential details of the textiles used are summarized in Table 1.

The prepared textile materials were additionally characterized to reveal their structure and magnetic properties. The digital optical microscope (Z16 APO, Leica Microsystems Co., Wetzlar, Germany) was used to provide information on the appearance of the textiles after functionalization with magnetic fluids. The morphology and structure of pure (native, non-magnetic) and magnetically modified woven and nonwoven textile materials were studied by scanning electron microscopy (SEM). Samples were analyzed using a Hitachi SU6600 scanning electron microscope (Hitachi Inc., Tokyo, Japan) with accelerating voltages of 1.0 or 1.5 kV. Using the same equipment, energy-dispersive X-ray spectroscopy (EDS) measurements were performed to investigate the content of the samples. A vibrational sample magnetometer (VSM) installed on a superconducting magnet (Cryogenic Ltd., London, UK) measured the magnetization curve at 295 K. Results are presented in Figure 2.

### 4.2. Preparation of Tissue-Mimicking Phantoms

The heating efficiency of magnetic textiles was tested using agar-based tissue-mimicking phantoms that imitate the properties of human tissues crucial for further biomedical applications. As we showed previously [27], the density, acoustic attenuation coefficient, and thermal conductivity of agar-based phantoms fall within the range of human tissues. In this study, we prepared phantoms with the mass concentration of agar powder 7% (*w*/*w*), which corresponds to the stiffness of tumors that is usually greater than the surrounding healthy tissues [28]. We prepared two types of agar-based phantoms: magnetic (MG phantom) containing 0.33% (*w*/*w*) of magnetic nanoparticles and nonmagnetic (pure phantom) ones. The preparation method consisted of several steps [19]. The distilled water, agar powder (plate count agar, product no. 88588, Sigma-Aldrich Co.), and magnetic nanoparticles (the same as the ones used to functionalize textiles, referring only to the results shown for magnetic phantoms in Figure 5A–D), were weighed with an accuracy of 0.1 mg. Boiling water was poured into a beaker containing agar powder (and magnetic powder, for magnetic phantoms only) to dissolve the powder. All ingredients were mixed for 1 min using an ultrasonic homogenizer (Sonoplus HD 3100, Bandelin GmBH, Berlin, Germany) equipped with a tapered tip (KE 76, length 176 mm and diameter 6 mm). Subsequently, the whole mixture was poured into small cylindrical beakers with an inner diameter of 1 cm, the height of 2.5 cm, and volume of around 3 mL. The phantoms were left at room temperature for 24 h to cool down and solidify through a gelation process. After solidification, the phantoms were removed from the glass beakers. Figure S1 presents the images of the prepared phantoms without and with a layer of a magnetic and a nonmagnetic textile.

### 4.3. Measurements of Temperature Increase in Tissue-Mimicking Phantoms under an Alternating Magnetic Field

When placed in the alternating magnetic field, magnetic nanoparticles act as a heat source due to the magnetic energy losses caused by relaxation and hysteresis mechanisms. For the alternating magnetic field generation, we used the induction heating system EASYHEAT provided by Ambrell Co. (New York, NY, USA). The frequency of the time-varying magnetic field was around 356 kHz, and the intensity of the field was 16.2 kA/m. The magnetic coil was water-cooled by the external cooling system.

**Figure 7:**
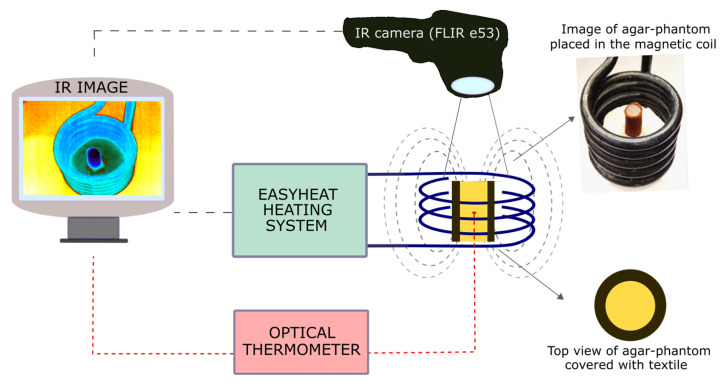
A scheme of the experimental setup for measurements of calorimetric effect under an alternating magnetic field.

To investigate the heating abilities of magnetic textiles, small pieces of textiles (1 × 1 cm) were placed inside the geometrical center of a heating coil. The temperature sensor FLUOTEMP provided by Photon Control Inc., Burnaby, BC, Canada, equipped with optic fiber (FTP-NY2) was placed below the material to record the temperature increase generated by the textile. To measure the temperature change inside the tissue-mimicking samples, we placed the same temperature sensor FLUOTEMP in the middle of the agar phantom. To investigate the temperature distribution of the phantom, depending on the radial distance from the heating textile to the sample center, we used an infrared (IR) camera (FLIR e53, FLIR Co., Wilsonville, OR, USA). The thermographic measurements are based on a so-called radiometric equation. The camera measures the radiance emitted by a tested object after considering the influence of radiance of the surrounding medium [29]. The IR camera was placed at a safe distance from the coil to ensure no interference between the camera sensor and the magnetic field. The scheme of the experiments is presented in Figure 7. The results from the IR camera were analyzed with the FLIR Research Studio version 2.1.0. Each experimental point presented in Figure 2, Figure 3, Figure 4, Figure 5, Figure 6 and Figure S2 was calculated based on the data obtained from several measurements and is shown with a standard deviation.

## 5. Conclusions

In this paper, we investigated four different textiles with magnetic nanoparticles embedded in their surface exposed to the alternating magnetic field. The temperature increase was determined in agar-based phantoms that mimicked the conditions in real tissues. Whilst the temperature elevations up to 50 °C were observed on the surface of the samples of textiles, the temperature measured inside phantoms was significantly lower and decreased radially from the phantoms’ edges to the center of the phantoms and depended on the concentration of the magnetic material within the textile, the textile’s structure, and the number of layers coating the phantom. Additionally, the synergetic effect leading to better magnetic heating efficiency was observed when the phantom also contained magnetic nanoparticles in its inside.

Our work showed that simple woven and non-woven textile materials can be a good starting point for a smart magnetic platform for hyperthermia measurements. We believe that our research opens a path for further testing of different textiles with various magnetic materials. Nanoparticles embedded in textiles, due to their high surface-to-volume ratio and simplicity of production via microwave applications, can act as carriers for drugs and molecules, e.g., anti-microbial molecules such as those presented for textile-based wound dressing supplemented by MNPs [30]. Moreover, the proposed magneto-responsive textiles could support other therapies, such as transdermal drug delivery. It was shown previously that magnetic heating can facilitate the penetration of drugs through the skin barrier [31]. However, magnetic textiles have not been proposed yet as such a delivery platform.

## Figures and Tables

**Table 1:** Description of textiles used in the study.

Abbreviation	Description	Concentration of Magnetite
MG TEX1	cotton-based ferrous sulfate solution dripped on a textile, followed by microwave treatment thickness: 0.5 mm	1.55 mg/cm^2^
MG TEX2	cotton-based immersion in ferrous sulfate solution, followed by microwave treatment thickness: 0.5 mm	1.23 mg/cm^2^
MG TEX3	acrylic felt-based immersion in microwave-synthesized MNP suspension thickness: 2 mm	2.62 mg/cm^2^
MG TEX4	acrylic felt-based immersion in microwave-synthesized MNP suspension modified by pressing in Thermo Press device thickness: 1 mm	2.62 mg/cm^3^

## Data Availability

Data will be made available on request.

## Supplementary Materials

The following supporting information can be downloaded at: https://www.mdpi.com/article/10.3390/ijms241411744/s1.
