# Supplementary material for: Magneto-Responsive Textiles for Non-Invasive Heating
Source: Int J Mol Sci. 2023 Jul 21;24(14):11744. doi: 10.3390/ijms241411744 (PMC10380502; doi:10.3390/ijms241411744)
Supplement: Supplementary file 1 [file ijms-24-11744-s001.zip › ijms-2498586-SI.pdf]

Electronic Supplementary Materials for:

Magneto-responsive textiles for non-invasive heating

*Arkadiusz Józefczak[1, *], Katarzyna Kaczmarek[1], Rafał Bielas[1], Jitka Procházková[2], Ivo Šafařík [2,3]*

[1]Faculty of Physics, Adam Mickiewicz University in Poznań,
Uniwersytetu Poznańskiego 2, 61-614 Poznań, Poland

[2]Department of Nanobiotechnology, Biology Centre, ISBB, Czech Academy of Sciences,
Na Sádkách 7, 370 05 České Budějovice, Czech Republic

[3]Regional Centre of Advanced Technologies and Materials, Czech Advanced Technology and Research Institute, Palacký University, Šlechtitelů 27, 783 71 Olomouc, Czech Republic

*e-mail: arkadiusz.jozefczak@amu.edu.pl

1.1. Tissue-mimicking phantoms covered with magnetic textiles

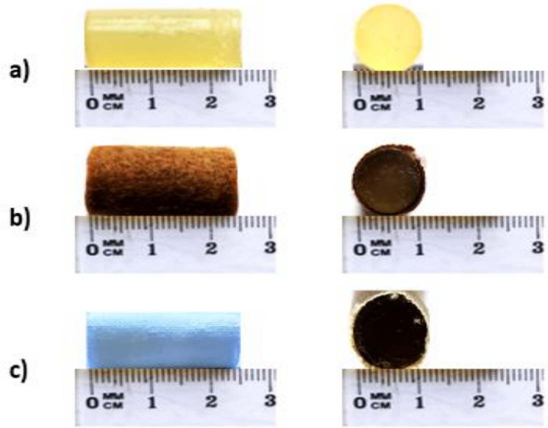

Figure S1 Digital photographs of different types of tissue-mimicking phantoms used in the study: a) pure agar-based phantom, b) agar-based phantom covered with magnetic textile no. 3 (MG TEX3) and c) agar-based phantom covered with pristine textile no. 1 (pure TEX1).

1.2. Temperature increase rate of textiles during magnetic heating

The temperature elevation obtained during magnetic heating depends on several factors, such as the type of magnetic textile (woven, non-woven) surrounding phantom, the concentration of magnetic nanoparticles incorporated into textiles, the number of used textile layers, and radial distance from the textile layer to the center of phantom. However, in terms of potential applications, not only the magnitude of the heating effect is crucial, but also the rate of temperature increase. **Figure S2** presents the comparison of the temperature increase rate (dT/dt) for all scenarios considered in our paper. Rates for **Figure S2a** were obtained from the linear fitting of temperature *versus* time curves obtained with optical thermometer for various number of textile layers, and the rates for **Figure S2b** were obtained from the linear fitting of temperature *versus* time curves of **Figure 6a** and **Figure 6b**, for the initial 15 seconds of the temperature rise.

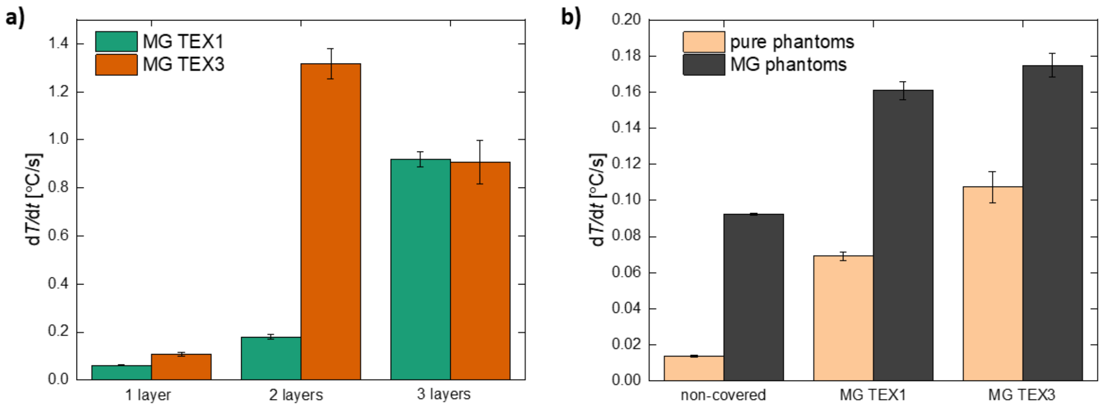

Figure S2 Temperature increase rate for magnetic heating under various experimental conditions. a) Comparison of the temperature elevation rate for various layers covering the pure phantom. b) Comparison of the temperature elevation rate for different textiles covering phantoms.

Figure S2a shows that the increase in the number of layers has a significant impact on the temperature increase rate, similarly to the temperature increase presented in **Figure 5a**. The presence of the second layer resulted in clearly more dynamic temperature elevation, especially when MG TEX3 was used. **Figure S2b** presents that regardless if MNPs are incorporated in pure agar phantoms or in textiles, they significantly impact the temperature increase rate. Additionally, it can be concluded that the type of used textile (woven MG TEX1, non-woven MG TEX3) is crucial for overall heating efficiency.
